# Is 2 h of Hypothermic Machine Perfusion for Pancreas Preservation Effective in Improving Graft Reperfusion?

**DOI:** 10.1097/TXD.0000000000001834

**Published:** 2025-07-24

**Authors:** Benoit Mesnard, Christophe Masset, Etohan Ogbemudia, Sarah Bruneau, Mohamed Elzawahry, Stéphanie Le Bas-Bernardet, David Minault, Jeremy Hervouet, Diego Cantarovich, Jérôme Rigaud, Lionel Badet, Peter Friend, Rutger Ploeg, Gilles Blancho, James Hunter, Thomas Prudhomme, Julien Branchereau

**Affiliations:** 1 Department of Urology and Transplantation Surgery, Nantes University Hospital, Nantes, France.; 2 Nantes Université, CHU Nantes1, INSERM, Centre for Research in Transplantation and Translational Immunology, UMR 1064, ITUN5, Nantes, France.; 3 Nuffield Department of Surgical Science, Oxford, United Kingdom.; 4 Department of Urology Surgery and Transplantation, Edouard Herriot Hospital, Lyon, France.

## Abstract

**Background.:**

Static cold storage (SCS) remains the standard method for organ preservation. The development of parenchymal edema during prolonged hypothermic machine perfusion (HMP) was a major barrier to the introduction of this technique for the preservation of pancreases. A short period of HMP could optimize the pancreas for reperfusion while minimizing the side effects related to perfusion. Our objective is to evaluate the impact of short-term HMP on the pancreatic reperfusion.

**Methods.:**

A preclinical study using a controlled donation after circulatory death porcine model was conducted. After procurement, the pancreases were preserved under hypothermic conditions for 2 h either by SCS (n = 4) or HMP (n = 4). After these 2 h of preservation, the pancreases were reperfused using a normothermic machine perfusion (NMP) for 2 h. During NMP, oxygenation, perfusion parameters, biochemical analyses, a glucose stimulation insulin secretion test, and an evaluation of ischemia/reperfusion injury by photoacoustic tomography were assessed.

**Results.:**

During NMP, resistance indices were significantly lower in the HMP group compared with the SCS group, even after 2 h of reperfusion. The tissue oxygen partial pressure was higher throughout NMP after HMP preservation. Lactate and amylase levels were equal between the 2 groups. Lipase levels were higher in the HMP group. The glucose stimulation test showed no difference between the 2 groups. Photoacoustic tomography assessment showed no endothelial damage in either group.

**Conclusions.:**

Our study suggests that a short-term HMP applied to pancreases for 2 h is effective in reducing resistance indexes and improving oxygenation.

## Graphical abstract

**Figure:**
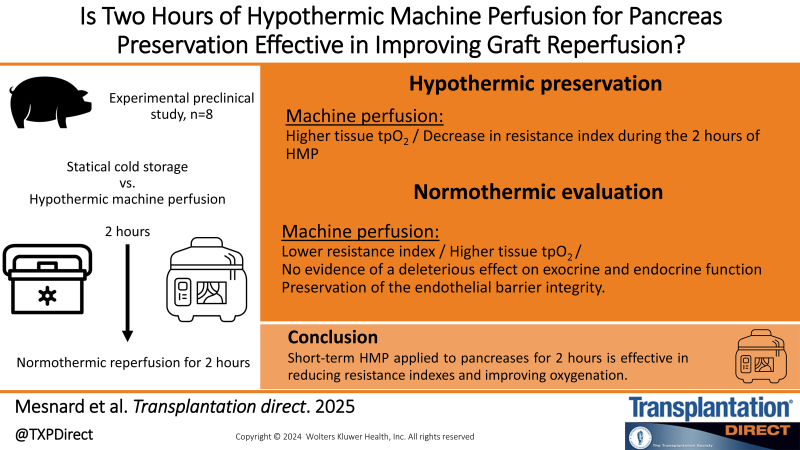

Pancreas transplantation (PT) is recognized as the most effective treatment for selected individuals with diabetes who have end-stage renal disease or brittle diabetes.^1^ PT provides excellent glycemic control, may reverse macrovascular and microvascular complications^2,3^ due to diabetes and corrects hypoglycemia unawareness. Studies have shown that PT enhances patients’ quality of life^4^ and, when performed in conjunction with renal transplantation, significantly increases patients’ life expectancy.^5^

In organ transplantation, ex vivo perfusion strategies (hypothermic machine perfusion [HMP], hypothermic oxygenated machine perfusion [HMPO_2_], normothermic machine perfusion [NMP]) have become established as reference preservation modalities. In other organs, some form of machine perfusion is the superior preservation method, with modalities adapted to each type of organ and the profile of the donors. In clinical pancreatic transplantation, static cold storage (SCS) remains the preservation standard regardless of the donor or preservation duration.^6^ Since the late 1980s and the advent of the Belzer solution in SCS,^7^ little evolution of static preservation modalities has occurred.^8^ Several teams from the 1970s to the 1980s focused on developing HMP for pancreas preservation using preclinical models. However, early experiments showed significant edema development within the pancreatic parenchyma during preservation. This edema was responsible for a higher transplant graft loss rate than after SCS preservation.^9-11^ Transplantation teams have managed to reduce edema during HMP by lowering perfusion pressures (20 mm Hg)^12^ and adapting perfusion solutions with a higher concentration of oncotic agents.^13^ It has been demonstrated in various preclinical models (porcine,^13,14^ nonhuman primate,^15^ and human^12^) that by adapting perfusion methods and protocols, it is possible to reduce its impact on pancreatic tissues after 24 h of HMP. However, the appearance of edema still seems inevitable. Despite many advancements, edema formation remains a major barrier to the clinical implementation of HMP. Therefore, it seems necessary to redefine the role of HMP and its usage modalities in clinical pancreatic transplantation.

Our team previously demonstrated that HMP in a 24-h preservation model allows for the preparation of pancreas reperfusion by reducing resistance indices (RIs) during reperfusion and increasing the oxygen available to the pancreas.^16^ Based on our observations, we have shown that RIs decrease during the first 2 h of HMP before stabilizing and even increasing due to edema. We hypothesize that short-term HMP preservation for 2 h would be sufficient to improve the reperfusion markers of pancreases. The aim of our study is to evaluate the impact of short-term HMP preservation compared with SCS on the reperfusion of pancreases.

## MATERIALS AND METHODS

### Experimental Protocol

**FIGURE 1.**
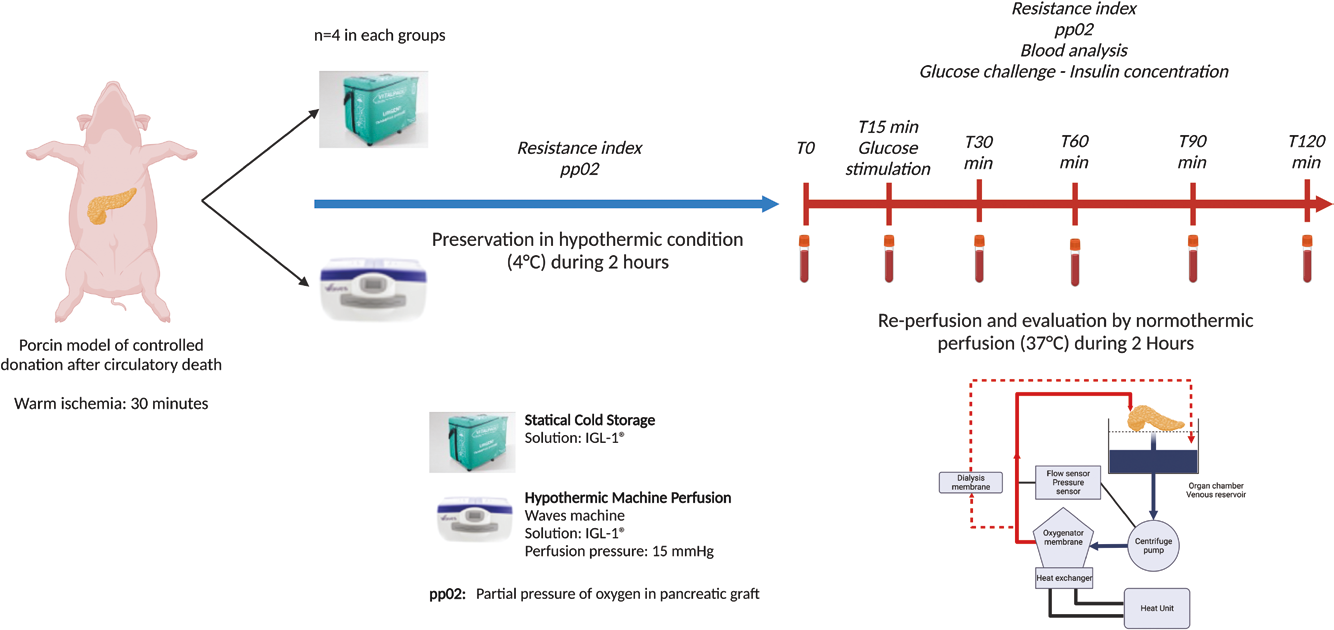
Experimental protocol. Porcine pancreases were procured in a cDCD model (30 min warm ischemia). Pancreases were then preserved under cold ischemia for 2 h according to statical cold storage or hypothermic machine perfusion. Finally, reperfusion and evaluation of the pancreas were conducted for 2 h using normothermic perfusion technique with autologous blood. cDCD, controlled donation after circulatory death.

This was a preclinical study using a porcine model (male Sus scrofa pigs, 80 kg, n = 8). A controlled donation after circulatory death (30 min of warm ischemia) pancreas procurement model was used according to a previously published technique by our team (Figure 1).^16,17^ After the pancreases were procured, a back-table preparation was performed for preservation. The pancreases were then preserved under hypothermic conditions for 2 h either in SCS (n = 4) or HMP (n = 4). After these 2 h of preservation, the pancreases were reperfused using an NMP technique for 2 h to assess the quality of pancreas reperfusion. During this period, perfusion data, biochemical analyses, a glucose stimulation test, and an evaluation of ischemia/reperfusion injury by photoacoustic tomography were performed at regular intervals.

### Pancreas Procurement and Preparation

Eight pancreases (4 in each group) were procured. After premedication in individual stalls, pigs were induced into general anesthesia using a combination of isoflurane (2%), nitrous oxide (49%), and oxygen (49%). Analgesia was administered as buprenorphine (0.05 mg/kg; Vétergesic, Sogeval, Laval, France) and paracetamol (25 mg/kg) via intravenous injection. A xyphopubic incision was made to expose the pancreas, followed by accessing the retroperitoneal vessels (aorta and subrenal vena cava). Before cannulating the aorta and vena cava, an intravenous bolus of unfractionated heparin (300 IU/kg) was administered. To induce in situ warm ischemia of the pancreas, the thoracic aorta was clamped after a diaphragmatic incision. Thirty minutes after clamping the aorta, abdominal organs were flushed at low pressure (20–40 cmH_2_O) with a fourth generation preservation solution cooled to 4 °C (osmotic agent: polyethylene glycol 35, 1 g/L; IGL-1, Institut Georges Lopez, Lissieu, France) via the aortic cannula. Crushed ice was introduced into the abdominal cavity to further cool the pancreas. Once organs were adequately flushed and clear venous return was observed through the vena cava, procurement was performed in accordance with human multiorgan procurement standards, with the pancreas procured en bloc with the aorta. The pancreas was prepared for perfusion, and aortic and portal cannulation were performed; leaks from lumbar arteries, hepatic artery, splenic artery, and root of the mesentery were ligated.

### Hypothermic Pancreas Preservation and Reperfusion

The pancreases were preserved under hypothermic conditions for 2 h using 2 different modalities. One group used SCS (n = 4) and the other group used HMP (n = 4). For both groups, we used the IGL-1 solution (Institut Georges Lopez). One liter of solution was used in each group for pancreas preservation. For the preservation of pancreases in HMP, we used the Waves Perfusion Machine (Institut Georges Lopez). This perfusion machine is CE marked for kidney HMP. Its cassette is adapted to the size of the pancreas. The perfusion is pulsatile at a fixed pressure with flow rate adjustment by the machine to meet the target pressure. All perfusions were performed at a systolic pressure of 15 mm Hg and a temperature of 4 °C. NMP was performed using a system derived from extracorporeal membrane oxygenation. The system comprises a centrifugal pump (Revolution centrifugal blood pump, Sorin Group, LivaNova, London, United Kingdom), an oxygenator (D100 oxygenator, Sorin Group), and a heat exchanger (HU 35 heater unit, Getinge, Sweden) to maintain the perfusate at a stable temperature of 37 °C. The circuit gas mixture (2 L/min) was composed of 95% oxygen and 5% carbon dioxide, delivered using an air/oxygen mixer (3500 series, Sechrist Industries, USA). The perfusate was made up of heparinized nonleukocyte-depleted autologous whole blood collected during organ procurement. The NMP circuit was primed with 1 L of this autologous blood and heated to 37 °C. Additives to the blood perfusate included 5000 units of heparin, 10 mg of nicardipine, and 1.2 g of co-amoxiclav. The perfusion pressure was regulated at 40 mm Hg. The NMP model has been previously described and has demonstrated its efficiency in preclinical models.^18^

### Perfusion Data and Biochemical Analyses

Measurement of pressure, flow, and RIs was performed throughout the normothermic perfusion. Tissue oxygen partial pressure (TpO_2_) in the parenchyma was measured. TpO_2_ was measured using the OxyLite Pro XL system (Oxford Optronix Ltd, Abingdon, United Kingdom). This measurement was enabled by probes the size of optical fibers (250 μm) introduced into the head and tail of the pancreas.^16^ The probes allow continuous analysis of TpO_2_, which is expressed in millimeters of mercury. The probes are introduced to a depth of 1 cm into the parenchyma and secured to the tissue to prevent any displacement during the experimentation. The complete methodology has been previously described.^16^ Continuous measurement was performed during hypothermic preservation and NMP reperfusion. Lactate, amylase, and lipase levels were measured in portal vein effluent sampled every 30 min during NMP, using an automated system operated by the biochemical analysis laboratory at Nantes University Hospital.

### Glucose Challenge During Normothermic Reperfusion

Fifteen minutes after the initiation of NMP, a glucose-stimulated insulin secretion test was conducted. A bolus of 50 mL of 5% glucose solution (2.5 g of glucose, 13.5 mM) was injected into the arterial side of the NMP circuit. Blood samples were collected at T0, T15 min (bolus), then every 2 min for 15 min, and subsequently every 30 min The blood samples were immediately centrifuged and aliquoted to recover the plasma. The plasma was then frozen with dry ice at –80 °C before storage. Insulin concentration was determined by ELISA (Porcine Insulin DuoSet ELISA kit R&D Systems Cat#DY8056-05, Minneapolis, MN).

### Photoacoustic Tomography Assessments of Ischemia/Reperfusion Injuries

An assessment of ischemia/reperfusion injuries was performed using photoacoustic tomography. The evaluation was conducted with a device from Deepcolor Imaging SAS (Nantes, France). It consists of a central unit and a detection head that can detect the presence of red blood cells in a 1 × 1 × 1 cm³ volume within the parenchyma, thereby assessing the pancreas vascularization. It also allows the evaluation of endothelial rupture indicated by the presence of hemoglobin outside the vessels. Detection was performed every 5 min for 30 min. The entire system has been detailed previously.^19^

### Ethical Approval

The research protocol (APAFiS no. 31507) received approval from the French Ministry of Research. All experiments were performed in accordance with the ARRIVE 2.0 guidelines^20^ and adhered to the European Directive 2010/63/EU regarding animal experimentation. Euthanasia procedures for the animals were carried out in compliance with legal regulations stipulated in the law on the protection of animals used for scientific purposes (conditions of killing: articles R214-98 to R214-98-1).

### Statistical Analysis

Statistical analyses were performed using GraphPad Prism software (version 9.1.1). Data are expressed as median ± interquartile range. To compare the parameters, a nonparametric 2-way test (Mann-Whitney) was performed by pooling all values over time. For the glucose-stimulated insulin secretion test, a nonparametric 2-way test (Mann-Whitney) was applied at T90min and T120 min.

## RESULTS

### TpO_2_ During Hypothermic Preservation and NMP Reperfusion

**FIGURE 2.**
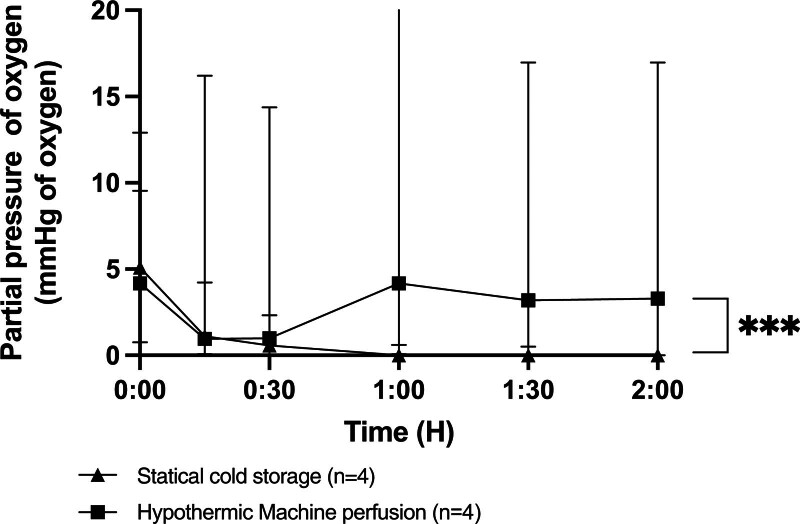
In tissue partial pressure of oxygen during hypothermic preservation according to static cold storage or hypothermic machine perfusion. Values expressed as median ± interquartile range. *P < 0.05; **P < 0.01; ***P < 0.001. ns, not significant.

**FIGURE 3.**
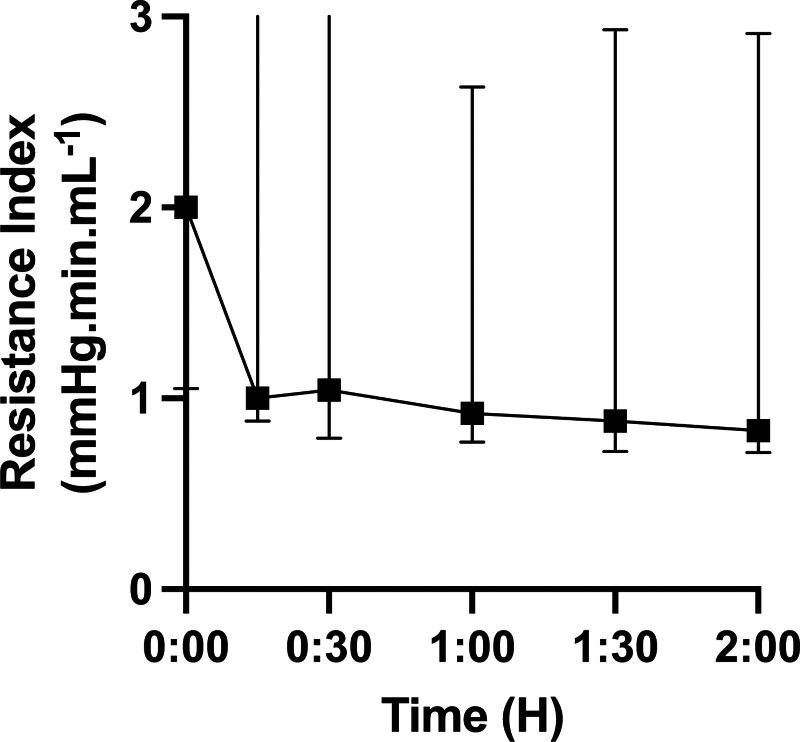
Resistance index during hypothermic preservation during hypothermic machine perfusion. Values expressed as median ± interquartile range. **P < 0.05; **P < 0.01; ***P < 0.001. ns, not significant.

**FIGURE 4.**
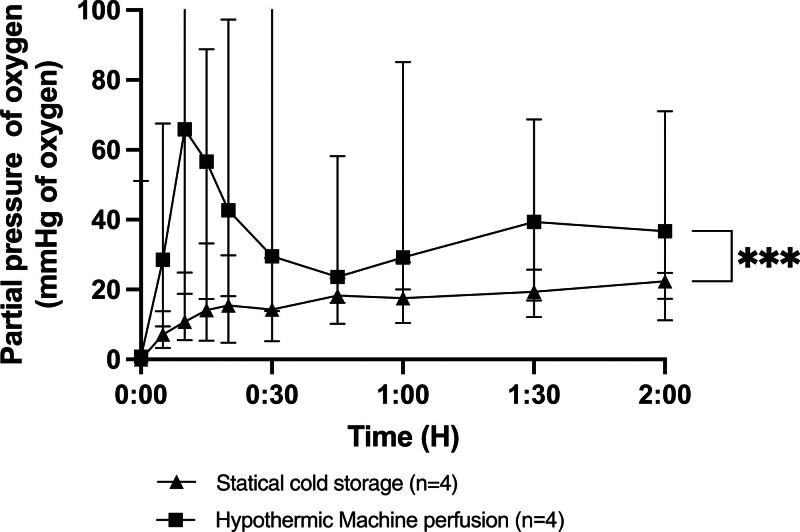
In tissue partial pressure of oxygen during normothermic reperfusion with normothermic machine perfusion. Values expressed as median ± interquartile range. *P < 0.05; **P < 0.01; ***P < 0.001. ns, not significant.

During hypothermic preservation with SCS, the TpO_2_ gradually decreased during the 2-h preservation period, ultimately reaching 0 mm Hg. In the HMP group, TpO_2_ remained stable throughout the preservation, maintaining around 5 mm Hg. There was a significant difference in oxygenation during preservation between SCS and HMP (Figure 2). A decrease in RIs was observed during the 2 h of HMP (Figure 3). During NMP, the TpO_2_ of pancreases after HMP remained higher throughout the 2-h reperfusion period, with an average value of around 35 mm Hg (in vivo oxygenation: 28 mm Hg^16^). In the SCS group, the oxygen available to the parenchyma was <20 mm Hg. A significant difference in perfusion was observed throughout the perfusion period (Figure 4).

### RIs During NMP

**FIGURE 5.**
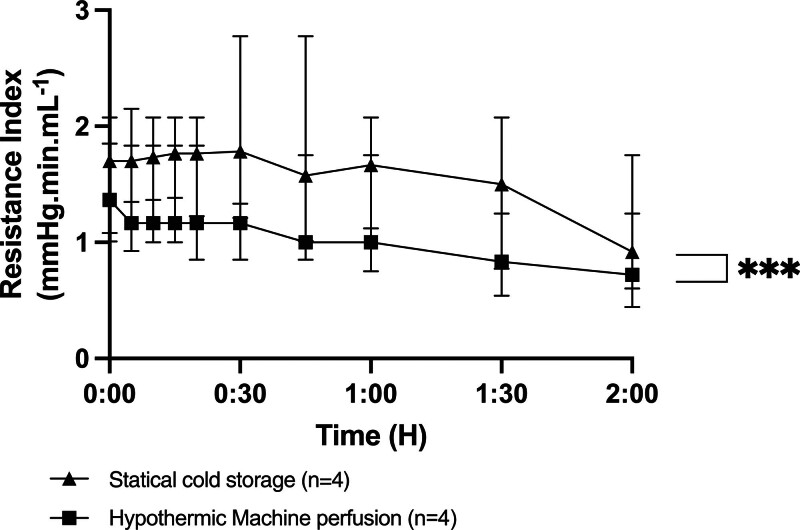
Resistance index during normothermic reperfusion with normothermic machine perfusion. Values expressed as median ± interquartile range. *P < 0.05; **P < 0.01; ***P < 0.001. ns, not significant.

A gradual but slow decrease in RIs is observed during NMP in both groups. The RIs were significantly higher in the SCS group. After 2 h of NMP, the RIs were 1 in the HMP group compared with 1.3 in the SCS group (Figure 5).

### Biochemical Analyses (Lactate, Lipase, Amylase)

**FIGURE 6.**
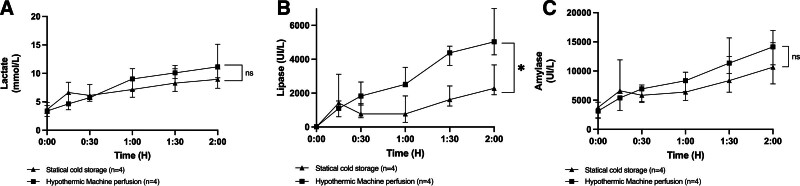
Concentration of lactate (A), lipase (B), and amylase (C) during normothermic reperfusion with normothermic machine perfusion. Values expressed as median ± interquartile range. *P < 0.05; **P < 0.01; ***P < 0.001. ns, not significant.

A progressive increase in the concentration of lactate, lipase, and amylase was observed throughout the NMP. There was no significant difference in the concentration of lactate and amylase between SCS and HMP preservation. Conversely, there was a significantly higher concentration of lipase during HMP (Figure 6).

### Glucose Challenge During Normothermic Reperfusion

**FIGURE 7.**
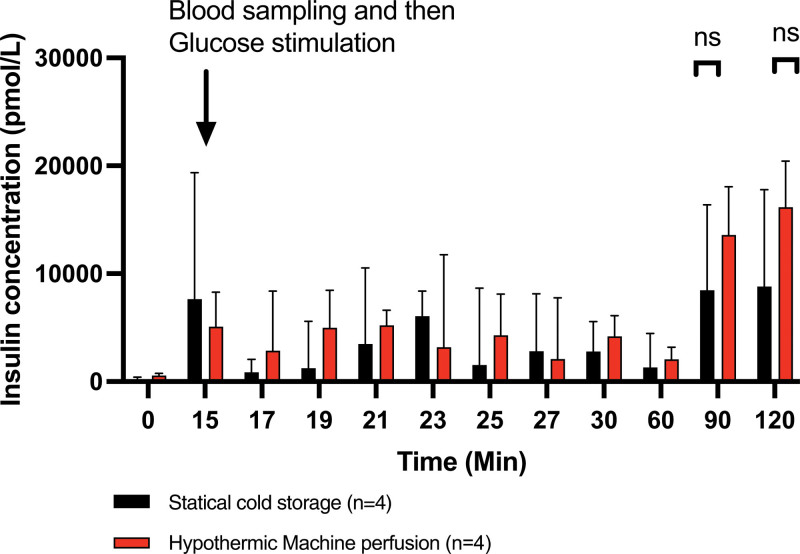
Insulin concentration after glucose stimulation (13.5 mM) at 15 min during normothermic reperfusion. Values expressed as median ± interquartile range. *P < 0.05; **P < 0.01; ***P < 0.001. ns, not significant.

Eight minutes after the glucose injection, an increase in insulin secretion was observed. After this peak, insulin concentration decreased between 23 and 60 min. At 90 and 120 min, elevated insulin concentrations were noted, likely due to the accumulation of insulin in the bloodstream over time during perfusion. Throughout the perfusion, no difference was observed between SCS and HMP (Figure 7). At T17 minutes, a drop in insulin concentration is observed in the SCS group. This appears to be a sampling artifact, as pancreatic blood flow in SCS is low, and the injected G5 volume is high, leading to a diluted blood sample.

### Photoacoustic Tomography Assessments of Ischemia/Reperfusion Injuries

**FIGURE 8.**
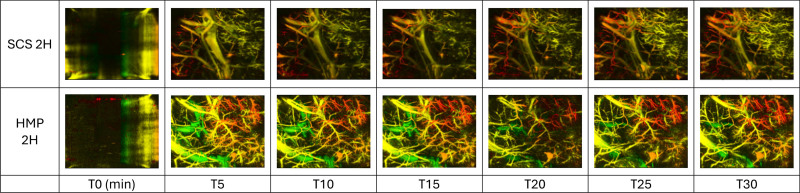
Photoacoustic tomography assessment of pancreases preserved for 2 h before normothermic reperfusion. The photoacoustic signal intensity indicates the amount of hemoglobin. Different colors represent measurement depth, aiding in the visualization of the 3-dimensional structure. HMP, hypothermic machine perfusion; SCS, static cold storage.

Photoacoustic tomography during NMP showed preservation of the vascular endothelium integrity in all 8 pancreases assessed. Rapid revascularization of the pancreas was observed, with almost complete revascularization within the first 5 min. A few extravasation lesions were noted (Figure 8). There appeared to be slightly faster reperfusion in pancreases preserved by HMP although quantitative data are lacking. This is demonstrated by a signal intensity that becomes visible more quickly and is more pronounced in the pancreases preserved by HMP.

## DISCUSSION

To date, the preservation of pancreases relies solely on SCS. Similar to other abdominal grafts, improving pancreas preservation may depend on the introduction of innovative perfusion methods such as hypothermic or normothermic perfusion. Pancreas perfusion faces 2 main challenges: the development of interstitial edema within the pancreatic parenchyma and the management of pancreatic enzymes during perfusion. Therefore, there is a need to adapt perfusion modalities to accommodate the specific requirements of pancreases.^21^

Since the first experiments in the 1980s, the occurrence of edema within the pancreatic parenchyma has been the primary issue associated with perfusion.^22^ Adjusting perfusion pressures during HMP by lowering pressures has helped limit edema.^22^ Additionally, adapting perfusion solutions to include higher concentrations of oncotic agents appears to be an effective method in reducing pancreases edema.^13^ However, the development of edema during perfusion is inevitable as the perfusion duration increases. Although this edema predominantly affects the interstitium rather than the pancreatic lobules, it remains a significant obstacle to the use of HMP.^12,13^ Our study suggests that a short 2-h HMP is sufficient to achieve the benefits associated with HMP, such as capillary opening, reduced RIs, and oxygen delivery to the pancreas. We previously demonstrated that a 24-h HMP versus a 24-h SCS allowed for better pancreas vascularization.^16^ The results between a 2-h HMP and a 24-h HMP seem comparable in terms of RIs and oxygenation on revascularization when indirectly compared with our previously described cohort of 24-h HMP,^16^ with no macroscopic edema observed in any of the pancreases preserved for 2 h. It is particularly interesting to note that the difference in resistance after 2 h of reperfusion remains significant over time and that the benefit of HMP persists during the initial hours of HMP. When examining resistance and tissue oxygenation during HMP, the data suggest that an even shorter perfusion duration of 1 h may be sufficient to prepare the pancreas for reperfusion. Our findings suggest the safety of short-term HMP, with similar lactate and amylase levels, glucose-stimulated insulin responses (beta-cell function), and photoacoustic tomography assessments showing no evident vascular damage.^19^ Only an increase in lipase levels was noted after HMP. The progressive increase in enzyme levels during NMP, as well as during HMP in our previous experiments, raises the necessity of enzyme clearance, as these enzymes can be damaging to the parenchyma. Protocols evaluating long-term NMP for pancreases agree on the need to include dialysis in the circuit to manage both edema and enzymes.^23,24^ Adding such a system to an HMP circuit could be a promising research avenue to optimize hypothermic perfusion during extended periods.

The implementation of a short HMP raises the question of its introduction into clinical practice. To date, in simultaneous pancreas-kidney transplants, there is no consensus on the sequence of organ transplantation. Most centers perform the pancreas transplant first to minimize the cold ischemia time of the pancreas as much as possible. However, some data in the literature suggest that performing a kidney-then-pancreas sequence transplant is feasible and may even be beneficial. Hau et al,^25^ in a retrospective single-center cohort, demonstrated fewer cases of pancreatitis, fewer venous thromboses, and fewer rejections within 3 mo in patients who underwent kidney transplantation first. This could be due to the effects of hemodynamics, systemic inflammation, and immunological responses on the pancreas in cases where the kidney transplant is performed secondarily. The implantation time of the kidney could be an ideal period to introduce a short HMP (2 h) as the durations are relatively similar. Thus, the kidney-then-pancreas sequence could be beneficial if a short HMP is introduced. However, these data are exploratory, and other patient cohorts suggest an advantage of the pancreas-then-kidney sequence in simultaneous kidney-pancreas transplantation.^26^

The second advantage of a 2-h HMP is its clinical applicability. Indeed, performing short perfusion is easier to implement than long perfusion in transplant centers, as it allows the procedure to be performed directly at the transplant center, avoiding potential challenges associated with organ transportation during perfusion. It eliminates the need for diverting exocrine secretions, which is required even in the context of hypothermic preservation. Moreover, the 2-h HMP can be performed while adhering to the conventional cold ischemia time frames for pancreatic transplantation, which are traditionally recognized as needing to remain <12 h. Finally, these findings are also encouraging for pancreatic islet isolation. Previous studies have demonstrated the benefits of HMP for the oxygenation and viability of pancreatic islets, with a 6-h perfusion duration.^27^ Our data suggest that a shorter HMP duration may also be sufficient to facilitate pancreatic islet isolation.

Our study is the first to specifically evaluate the impact of short HMP (2 h) on the reperfusion of pancreases. These new data propose an approach for introducing HMP and encourage teams to implement short-term HMP for 2 h. Our study has several limitations. First, it does not account for the cold ischemia period that might precede short-term perfusion; indeed, a 2-h HMP requires additional preservation through SCS. Additionally, our system does not include a dialysis unit; thus, the interpretation of enzyme and insulin concentrations is subject to an accumulation bias in the perfusion solution. Furthermore, there is no quantitative analysis of photoacoustic tomography, and our study also lacks histological analysis and wet-to-dry ratio analysis to confirm the absence of interstitial or lobular edema after a 2-h perfusion. Additionally, the use of nicardipine to induce vasodilation and facilitate pancreatic perfusion during NMP could potentially influence insulin secretion (as a calcium channel blocker) and affect the insulin test. Ultimately, despite performing an insulin test during NMP, we did not proceed with the isolation and culture of pancreatic islets, which would have allowed for in vitro endocrine function testing.

In conclusion, our study demonstrates that short-term HMP applied to the pancreas does not cause harm and appears to be effective in reducing RIs while improving oxygenation during 2-h perfusion.

## ACKNOWLEDGMENTS

The authors thank the French National Agency (Agence de la biomedicine) for grant funding, the French Urological Association (Association Française d’Urologie) for grant funding, the Nantes University Hospital for grant funding, the biochemical analysis laboratory of the Nantes University Hospital for logistical support, the Institut Georges Lopez for grant funding and technical support, and the Deepcolor Imaging SAS (Nantes, France, http://www.deepcolorimaging.com) for providing the platform, the time dedicated by their technical teams, and their scientific expertise.

## References

[1] Boggi U Vistoli F Marchetti P; World Consensus Group on Pancreas Transplantation. First world consensus conference on pancreas transplantation: part I-methods and results of literature search. Am J Transplant. 2021;21(Suppl 3):1–16. 10.1111/ajt.16738 PMC8519053 34245116

[2] Ziaja J Kolonko A Kamińska D. Long-term outcomes of kidney and simultaneous pancreas-kidney transplantation in recipients with type 1 diabetes mellitus: silesian experience. Transplant Proc. 2016;48:1681–1686. 27496471 10.1016/j.transproceed.2016.01.082

[3] Pérez Tamajón L Marrero Miranda D Caballero Figueroa A. Improved cardiovascular risk profile of patients with type 1 diabetes mellitus and renal failure after simultaneous pancreas–kidney transplantation. Transplant Proc. 2005;37:3979–3980. 16386603 10.1016/j.transproceed.2005.09.156

[4] Rajkumar T Mazid S Vucak-Dzumhur M. Health-related quality of life following kidney and simultaneous pancreas kidney transplantation. Nephrology (Carlton). 2019;24:975–982. 30393905 10.1111/nep.13523

[5] Gruessner AC Gruessner RW. Pancreas transplantation of US and non-US cases from 2005 to 2014 as reported to the United Network for Organ Sharing (UNOS) and the International Pancreas Transplant Registry (IPTR). Rev Diabet Stud. 2016;13:35–58. 26982345 10.1900/RDS.2016.13.35 PMC5291181

[6] Branchereau J Hunter J Friend P. Pancreas preservation: clinical practice and future developments. Curr Opin Organ Transplant. 2020;25:329–335. 32618717 10.1097/MOT.0000000000000784

[7] Ploeg RJ Goossens D Sollinger HW. Efficacy of 48-hour pancreas preservation with UW solution in the dog allograft model. Transplant Proc. 1988;20:1026–1028. 3055478

[8] Belzer FO D’Alessandro AM Hoffmann RM. The use of UW solution in clinical transplantation. A 4-year experience. Ann Surg. 1992;215:579–583; discussion 584. 1632679 10.1097/00000658-199206000-00004 PMC1242507

[9] Florack G Sutherland DE Heil J. Preservation of canine segmental pancreatic autografts: cold storage versus pulsatile machine perfusion. J Surg Res. 1983;34:493–504. 6341715 10.1016/0022-4804(83)90101-4

[10] Tersigni R Toledo-Pereyra LH Pinkham J. Pancreaticoduodenal preservation by hypothermic pulsatile perfusion for twenty-four hours. Ann Surg. 1975;182:743–748. 1103762 10.1097/00000658-197512000-00016 PMC1343973

[11] Brynger H. Twenty-four-hour preservation of the duct-ligated canine pancreatic allograft. Eur Surg Res. 1975;7:341–354. 1102314 10.1159/000127819

[12] Branchereau J Renaudin K Kervella D. Hypothermic pulsatile perfusion of human pancreas: preliminary technical feasibility study based on histology. Cryobiology. 2018;85:56–62. 30292812 10.1016/j.cryobiol.2018.10.002

[13] Ogbemudia AE Hakim G Dengu F. Development of ex situ normothermic reperfusion as an innovative method to assess pancreases after preservation. Transpl Int. 2021;34:1630–1642. 34448276 10.1111/tri.13990

[14] Elzawahry M Fallon J Nawaz S. Oxygenated hypothermic machine perfusion of the pancreas; a porcine circulatory death model to compare a ‘continuous’ and an ‘end-ischaemic’ approach. Transplantation. 2023;107(10S2):146–147.

[15] Prudhomme T Renaudin K Lo Faro ML. Ex situ hypothermic perfusion of nonhuman primate pancreas: a feasibility study. Artif Organs. 2020;44:736–743. 31995645 10.1111/aor.13655

[16] Mesnard B Ogbemudia E Bruneau S. Pancreas preservation: hypothermic oxygenated perfusion to improve graft reperfusion. Transplantation. 2024;109:e1–e10. 39656523 10.1097/TP.0000000000005111

[17] Prudhomme T Kervella D Ogbemudia AE. Successful pancreas allotransplantations after hypothermic machine perfusion in a novel diabetic porcine model: a controlled study. Transpl Int. 2021;34:353–364. 33275807 10.1111/tri.13797

[18] Mesnard B Kervella D Prudhomme T. Pancreas ex-situ preservation and evaluation. Development of a normothermic machine perfusion system. Eur J Transplant. 2022;1:56–62.

[19] Mesnard B Branchereau J Prudhomme T. Photoacoustic tomography assessments during ex vivo normothermic perfusion. A novel and noninvasive modality to evaluate endothelial integrity. Transplantation. 2025;109:e343–e344. 39932762 10.1097/TP.0000000000005342

[20] Percie du Sert N Hurst V Ahluwalia A. The ARRIVE guidelines 2.0: updated guidelines for reporting animal research. PLoS Biol. 2020;18:e3000410. 32663219 10.1371/journal.pbio.3000410 PMC7360023

[21] Ferrer-Fàbrega J Mesnard B Messner F. European Society for Organ Transplantation (ESOT) consensus statement on the role of pancreas machine perfusion to increase the donor pool for beta cell replacement therapy. Transpl Int. 2023;36:11374. 37547751 10.3389/ti.2023.11374 PMC10402633

[22] Prudhomme T Kervella D Le Bas-Bernardet S. Ex situ perfusion of pancreas for whole-organ transplantation: is it safe and feasible? A systematic review. J Diabetes Sci Technol. 2020;14:120–134. 31409133 10.1177/1932296819869312 PMC7189158

[23] Parmentier C Ray S Mazilescu LI. Normothermic ex vivo machine perfusion of discarded human pancreas allografts: a feasibility study. Transpl Int. 2023;36:10936. 37252614 10.3389/ti.2023.10936 PMC10210159

[24] Ray S Parmentier C Kawamura M. Reanimating pancreatic grafts subjected to prolonged cold ischemic injury using normothermic ex vivo perfusion. Transplant Direct. 2024;10:e1620. 38617463 10.1097/TXD.0000000000001620 PMC11013695

[25] Hau HM Jahn N Rademacher S. The value of graft implantation sequence in simultaneous pancreas-kidney transplantation on the outcome and graft survival. J Clin Med. 2021;10:1632. 33921391 10.3390/jcm10081632 PMC8070486

[26] Niclauss N Bédat B Morel P. Impact of graft implantation order on graft survival in simultaneous pancreas-kidney transplantation. Transpl Int. 2016;29:627–635. 26987785 10.1111/tri.12773

[27] Doppenberg JB Leemkuil M Engelse MA. Hypothermic oxygenated machine perfusion of the human pancreas for clinical islet isolation: a prospective feasibility study. Transpl Int. 2021;34:1397–1407. 34036616 10.1111/tri.13927 PMC8456912

